# An Ultrasonic Microrobot Enabling Ultrafast Bidirectional Navigation in Confined Tubular Environments

**DOI:** 10.1007/s40820-025-01894-y

**Published:** 2025-08-25

**Authors:** Meng Cui, Liyun Zhen, Xingyu Bai, Lihan Yu, Xuhao Chen, Jingquan Liu, Qingkun Liu, Bin Yang

**Affiliations:** 1 National Key Laboratory of Advanced Micro and Nano Manufacture Technology, Shanghai Jiao Tong University, Shanghai, 200240 People’s Republic of China; 2 Department of Micro/Nano Electronics, School of Electronic Information and Electrical Engineering, Shanghai Jiao Tong University, Shanghai, 200240 People’s Republic of China

**Keywords:** Ultrasonic microrobot, Piezoelectric composite film microstructure, MEMS fabrication, Bidirectional locomotion, Confined pipeline inspection

## Abstract

Pipelines are extensively used in environments such as nuclear power plants, chemical factories, and medical devices to transport gases and liquids. These tubular environments often feature complex geometries, confined spaces, and millimeter-scale height restrictions, presenting significant challenges to conventional inspection methods. Here, we present an ultrasonic microrobot (weight, 80 mg; dimensions, 24 mm × 7 mm; thickness, 210 μm) to realize agile and bidirectional navigation in narrow pipelines. The ultrathin structural design of the robot is achieved through a high-performance piezoelectric composite film microstructure based on MEMS technology. The robot exhibits various vibration modes when driven by ultrasonic frequency signals, its motion speed reaches 81 cm s^−1^ at 54.8 kHz, exceeding that of the fastest piezoelectric microrobots, and its forward and backward motion direction is controllable through frequency modulation, while the minimum driving voltage for initial movement can be as low as 3 V_P-P_. Additionally, the robot can effortlessly climb slopes up to 24.25° and carry loads more than 36 times its weight. The robot is capable of agile navigation through curved L-shaped pipes, pipes made of various materials (acrylic, stainless steel, and polyvinyl chloride), and even over water. To further demonstrate its inspection capabilities, a micro-endoscope camera is integrated into the robot, enabling real-time image capture inside glass pipes.

**Supplementary Information:**

The online version contains supplementary material available at 10.1007/s40820-025-01894-y.

## Highlights

- An ultrasonic microrobot achieves bidirectional high-speed locomotion (81 cm s^−1^) in micro-pipes via frequency modulation.
- MEMS-fabricated ultrathin piezoelectric composite film enables rapid navigation within confined pipeline (4 mm height), slope climbing (24.25°), and notable load-carrying (>36 times its weight).
- The microrobot demonstrates agile locomotion across curved pipes, pipes made of various materials, and even over water; integrated micro-endoscope camera enables real-time imaging, highlighting great potential for efficient pipeline inspection.

**Supplementary Information:**

The online version contains supplementary material available at 10.1007/s40820-025-01894-y.

## Graphical abstract

**Figure:**
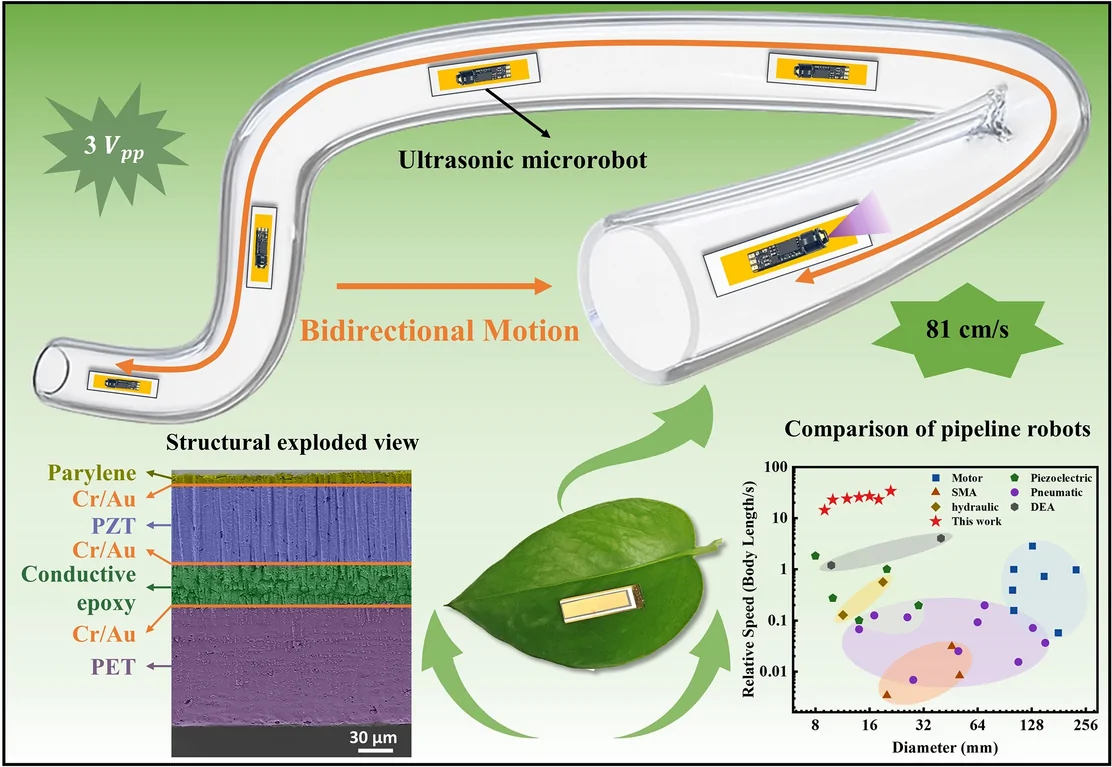

## Introduction

Pipelines are widely used in industries such as petrochemicals, power generation, nuclear energy, and healthcare for transporting gases, liquids, and other critical substances [1]. Over time, these pipeline systems often face issues such as aging, corrosion, blockages, and damage, making regular internal inspections crucial [2]. These pipelines typically feature complex geometries and contain millimeter-scale confined spaces, presenting significant challenges for conventional inspection methods, which are often unable to access narrow and intricate spaces. In response to this challenge, microrobots, as an emerging technology, have demonstrated great potential in pipeline inspection in recent years [3]. Microrobots can access confined spaces that are difficult for humans to reach, enabling efficient internal inspections [4].

A variety of microrobots based on diverse actuation principles—including magnetic [5–13], pneumatic [14, 15], dielectric elastomer actuators (DEAs) [4, 16–18], electromagnetic [19, 20], electrochemical [21–24], light [25–28], and piezoelectric [29–36]—have been developed, demonstrating potential motion advantages in micro-pipe environments. However, requirements such as actuation mechanisms and their structural dimensions further limit the application environment. For example, microrobots driven by pneumatic or electromagnetic motors are typically limited to inspection tasks in pipes with relatively large diameters. Magnetic actuation may be unsuitable for ferromagnetic pipes, while light-driven robots face challenges operating in opaque pipes. Moreover, both types of actuations generally result in slow movement speeds. Although microrobots driven by DEAs have some speed advantages, they often require kilovolt-level power supplies. Consequently, it is a challenge to realize a low-voltage driven microrobot capable of rapid navigation in confined tubular environments.

In contrast, piezoelectric driving robots offer several advantages, including compact structure, high power density, fast response, high resolution, and immunity to electromagnetic interference [37]. Microrobots based on polyvinylidene fluoride (PVDF) have been successfully developed, demonstrating some flexibility. However, due to the low piezoelectric coefficient of functional materials, they still require relatively high driving voltages during motion and exhibit poor load-bearing capabilities [30]. Compared to PVDF, lead zirconate titanate (PZT) exhibits a much higher piezoelectric coefficient (more than 10 times that of PVDF), providing stronger performance at the same driving voltage. PZT has been widely used in microrobots and is expected to offer unique performance advantages in confined pipeline environments [38].

Piezoelectric microrobots can be broadly classified into two categories based on their driving frequency. The first category operates in the low-frequency range below 1 kHz. This type of piezoelectric actuator primarily works in low-order vibration modes with large amplitudes. However, achieving stable and rapid motion requires a well-designed leg structure, as these robots typically demonstrate a jumping mechanism similar to animal locomotion [29, 32, 39]. This approach proves inadequate for navigation within millimeter-scale narrow pipes and is further hindered by susceptibility to environmental noise, which compromises operational stability. The second category operates at ultrasonic frequencies, with frequencies exceeding 20 kHz. In this range, the vibration amplitudes of piezoelectric actuators are obviously smaller than that in low-frequency operation, and robots leverage complex high-order vibration modes. Near its resonant frequency, the robot readily excites its primary bending mode, allowing surface nodes to trace larger elliptical trajectories within each cycle. Simultaneously, ultrasonic actuation introduces kilohertz-scale micro-vibrations that periodically reduce the normal contact force, causing more frequent transitions from static to kinetic friction, markedly lowering interface friction and thereby further improving thrust utilization. These enable high-speed and stable motion without additional leg structures, and by adjusting the frequency, multimodal motion can be achieved. Additionally, ultrasonic operation circumvents challenges such as interference from environmental noise. Although several ultrasonic piezoelectric microrobots have been developed, their rigid structural designs have constrained their size to the centimeter-scale and increased their weight [34, 40, 41]. Consequently, navigating in confined environments, particularly millimeter-scale narrow pipes, remains a significant challenge.

In this work, inspired by the retrograde wave gait coupled motion of multi-legged centipedes, we present an ultrasonic microrobot for inspection tasks in confined tubular environments. The robot utilizes a composite film microstructure consisting of a thinned PZT film bonded to a flexible polyethylene terephthalate (PET) substrate via transfer printing, along with an encapsulation layer made of parylene-C film. Powered by ultrasonic frequency signals, the robot can navigate rapidly through narrow pipes with diameters ranging from 9 to 27 mm and achieve a maximum speed of 81 cm s^−1^, which is 57% faster than the fastest piezoelectric microrobots [42]. Its motion direction can be easily reversed by modulating the driving frequency, enabling agile bidirectional movement. Even in confined spaces with only 4 mm of height, the robot maintains a high navigation speed of 34.3 cm s^−1^. Moreover, the robot operates at a low driving voltage of 3 V_P-P_, two orders of magnitude lower than that required by DEAs. The robot also demonstrates exceptional performance in slope climbing and load-bearing capabilities, and can navigate in L-shaped pipes, pipes made of various materials and even over water. Furthermore, by integrating a micro-endoscope camera, the robot can perform real-time image capture inside glass pipes, demonstrating its potential for high-precision inspection in confined tubular environments.

## Experimental Section

### Preparation of the Microrobot

The robot was composed of a flexible PET substrate, a PZT thin film, and encapsulation materials. The fabrication process began by cutting a polarized PZT substrate (300 μm thickness, C-83H, Fuji Ceramics Co., Japan) into strips measuring 5 mm × 20 mm using a dicing saw (DAD3650 Dicing Saw, DISCO Corporation). The PZT strips were then thinned to 70 μm using a mechanical grinder (BG810 Grinder, DISCO Corporation). Next, a UV laser cutting machine (CF430UV, Wuhan Topwin Optoelectronics Technology Co., Ltd) was used to pattern PET film (100 μm thickness) into strips measuring 7 mm × 24 mm. Cr/Au electrodes (20/200 nm) were sputtered onto the surfaces of the thinned PZT strips and patterned PET films using a magnetron sputtering device (TRP-450, SKY Technology Development Co., Ltd). A 30 μm-thick layer of conductive silver epoxy resin (DAD-87, Shanghai Research Institute of Synthetic Resins Co., Ltd) was screen-printed onto the PET film surface. The thinned PZT strip, with its sputtered electrode side facing down, was aligned and placed onto the target position of the conductive silver epoxy layer. To provide a buffer layer, a PDMS film (Sylgard 184 silicone elastomer, 10:1) was applied to the top surface of the device. A 500 g square iron plate was then placed on top of the PDMS film, and the device was cured on a heating platform at 85 °C for 3 h. After curing, Cr/Au electrodes (20/200 nm) were sputtered onto the top surface of the PZT thin film. Silver electrode wires (50 μm diameter, 50 cm length, Kunshan Shengshi Jingxin New Material Co., Ltd) were attached to the top surfaces of the PZT and PET films using conductive silver epoxy resin. The device was further cured on a heating platform at 85 °C for an additional 3 h to ensure the electrode wires were securely connected. Finally, a parylene-C film (10 μm thickness) was deposited on the surface of the device via chemical vapor deposition to provide encapsulation.

### Characterization and Measurement

The cross sectional view of the microrobot was characterized using a scanning electron microscope (ULTRA PLUS, Carl Zeiss AG) at a magnification of 228 times. The driving signal for the microrobot was generated by a function waveform generator (33511B, Keysight Technologies Inc., USA), with its amplitude adjusted by a voltage amplifier (ATA-2022B, Xi'an Aigtek Electronic Technology Co., Ltd., China). The RMS operating current of the robot was measured with a high-precision digital multimeter (DMM6500, Keithley Instruments, Inc., USA), and the body temperature of the robot was monitored using an infrared camera (FOTRIC 228 s, FOTRIC Inc., China). The vibration velocity response spectrum at various frequencies, as well as the vibration mode at the resonant frequency, was measured using a Doppler laser vibrometer (PSV-500, Polytec GmbH, Germany).

### Finite Element Analysis

Finite element analysis (FEA) was performed using COMSOL Multiphysics 6.1 to determine the resonant frequency of the robot and vibration modes during forward and backward motion. The geometric model in COMSOL consisted primarily of PET, conductive silver epoxy resin, and PZT, with the effects of encapsulation and electrode materials omitted for simplification. Detailed material parameters are provided in Table S1. The solid mechanics and electrostatics modules were employed to analyze the mechanical and piezoelectric properties of the robot. To approximate real motion conditions, the mechanical boundary conditions were set as free. A voltage of 50 V was applied to the PZT material to investigate the vibration modes of the robot across different frequencies.

## Results and Discussion

### Design and Fabrication of Ultrasonic Microrobots

**Fig. 1:**
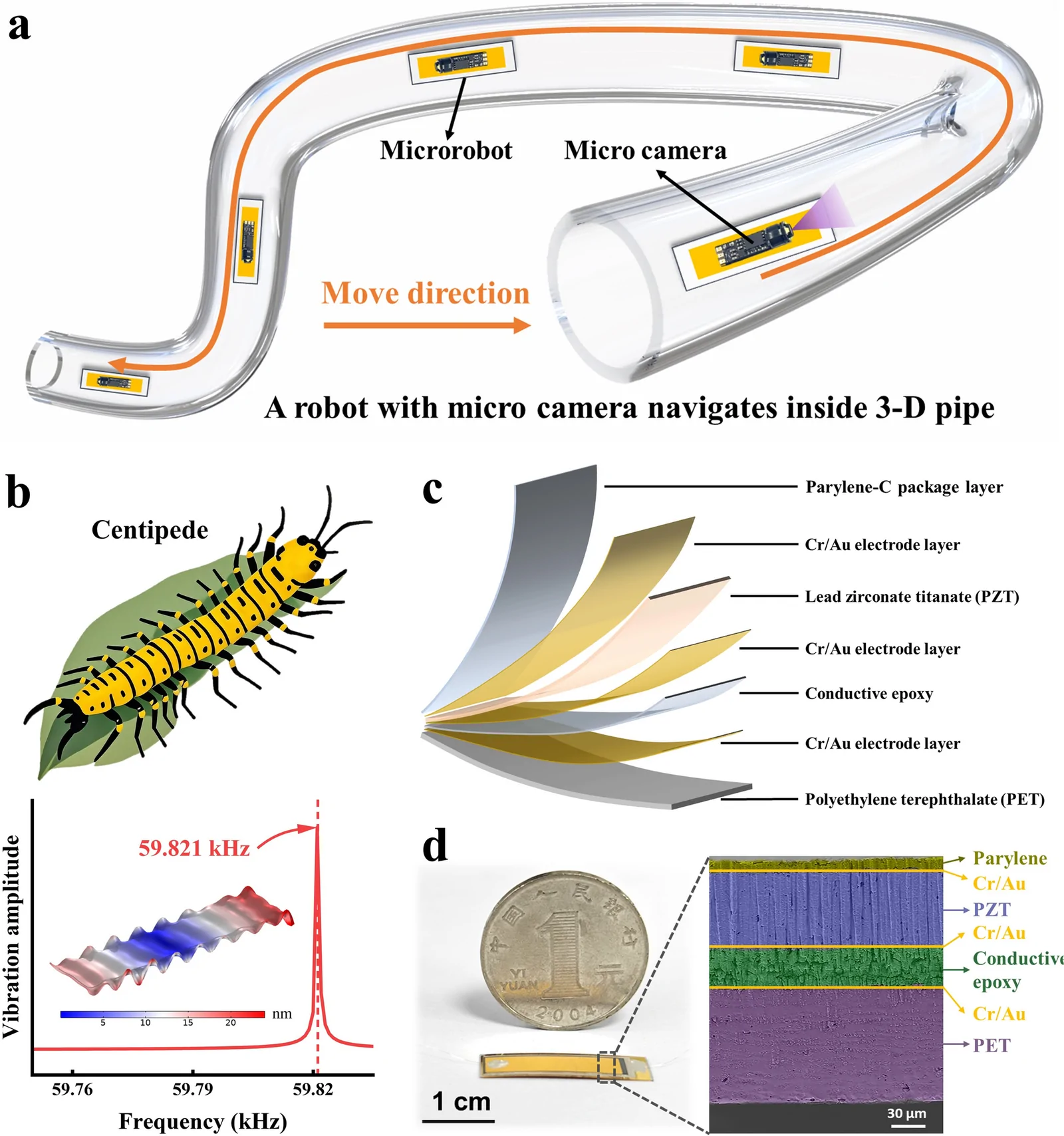
Design of the ultrasonic microrobot.** a** Conceptual illustration of the microrobot equipped with a micro-endoscope camera performing inspection tasks in a glass pipe. **b** Comparison between the picture of a centipede and the simulated working mode of the microrobot at its resonant frequency demonstrates that the robot exhibits a multi-legged locomotion mode similar to that of the centipede. **c** Exploded schematic diagram detailing the structural design of the microrobot. **d** Optical photograph showing the fabricated microrobot alongside a Chinese coin for size comparison, with an inset SEM image providing a cross sectional view of the microrobot, highlighting its multilayer material composition

The design of our ultrasonic microrobot is inspired by the retrograde wave gait coupled locomotion of multi-legged centipedes, enabling rapid navigation in micro-pipes, as shown in Fig. 1a. Figure 1b illustrates the simulated motion mode of the robot at an ultrasonic resonant frequency of 59.821 kHz. When an ultrasonic driving signal near the resonant frequency is applied, the robot exhibits a retrograde wave gait similar to that of a centipede, generating head-to-tail bending waves in resonant modes, with the net direction of motion opposite to the wave direction, consistent with the multi-legged locomotor gait pattern of a centipede [43] (Movie S1). The robot consists of three primary components: a flexible PET substrate, a PZT thin film, and a parylene-C encapsulation layer, as shown in Fig. 1c. The PZT thin film serves as the actuation unit, exhibiting periodic elongation and contraction under applied periodic driving signals. Combined with the flexible PET substrate, this enables controlled and efficient motion. Cr/Au electrode layers (20/200 nm) are sputtered onto the top surface of the PET substrate and the top/bottom surfaces of the PZT film. The PET substrate and PZT film are securely bonded using a conductive silver epoxy resin layer, followed by the deposition of a 10 μm-thick parylene-C film as encapsulation. Figure 1d shows the fabricated miniature robot alongside a standard Chinese coin for size comparison. The robot measures 24 mm in length, 7 mm in width, and 210 μm in thickness, with a total weight of 80 mg. The inset shows a cross sectional SEM view of the robot, revealing a 100 μm-thick PET substrate, a 30 μm-thick conductive silver epoxy bonding layer, a 70 μm-thick PZT film, three Cr/Au electrode layers (20/200 nm thick), and a 10 μm-thick parylene-C encapsulation layer.

The fabrication process of the robot is illustrated in Fig. S1 and consists of four primary steps: (1) mechanical thinning and patterning of the PZT substrate, (2) sputtering Cr/Au electrodes on the surface of the PET and PZT, (3) transfer bonding and Cr/Au electrode deposition on the top surface of the PZT, and (4) electrode wiring and parylene-C film encapsulation. The details are described in the Supplementary Materials. We measured the piezoelectric coefficient of the PZT thick film with a quasi-static piezoelectric meter, obtaining a d_33_ value of 248.5 pC N^−1^.

### Work Mechanism and Performance Characterization

**Fig. 2:**
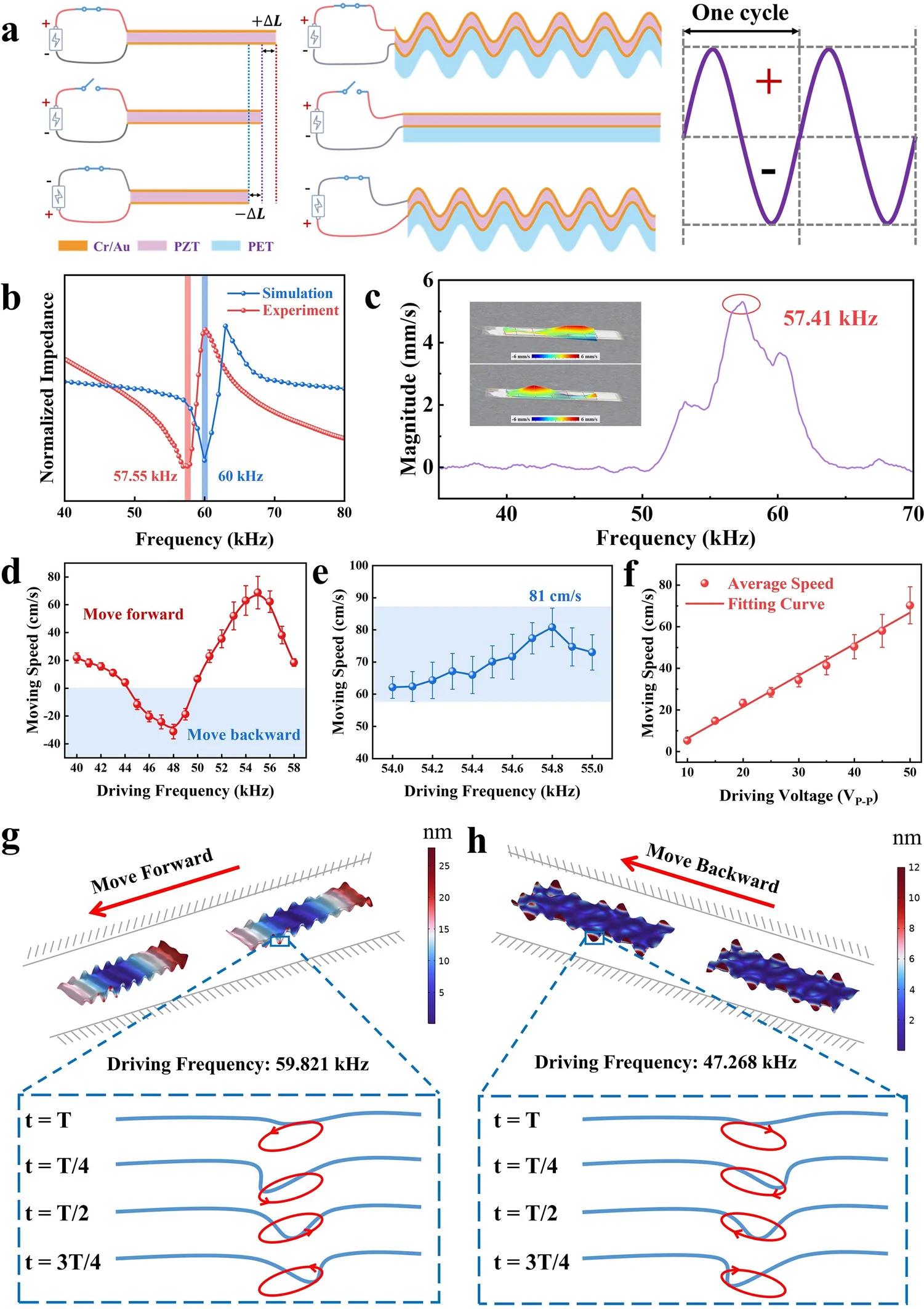
Motion mechanism and performance characterization of the ultrasonic microrobot. **a** The left image illustrates the actuation mechanism of the PZT thin film, where the film elongates by ∆L under positive voltage and contracts by ∆L under negative voltage. The right image depicts the corresponding actuation behavior of the microrobot when driven by a sinusoidal signal at ultrasonic frequency. **b** Comparison between the simulated and experimentally measured impedance characteristics of the microrobot. **c** Vibration test results were obtained using a laser Doppler vibrometer, showing the vibration velocity response spectrum as a function of frequency and the vibration mode at maximum vibration velocity. **d** Relationship between the movement speed of the microrobot and the driving signal frequency, with the shaded portion representing backward movement. **e** Fine-tuned test results of the maximum moving speed of the microrobot versus the driving signal frequency. **f** Relationship between the movement speed of the microrobot and the voltage amplitude of the driving signal. All error bars represent the standard deviation of three times of measurements. **g** Mechanistic analysis of the microrobot during forward motion, showing the simulated forward motion mode and the characteristic frequency of 59.821 kHz. The simplified diagram below illustrates that the nodes form counterclockwise elliptical trajectories with the contact surface. **h** Mechanistic analysis of the microrobot during backward motion, showing the simulated backward motion mode and the characteristic frequency of 47.268 kHz. The simplified diagram below illustrates that the nodes form clockwise elliptical trajectories with the contact surface

The actuation mechanism of the PZT thin film and the microrobot with a piezoelectric composite film microstructure is conceptually illustrated in Fig. 2a. As shown on the left side of Fig. 2a, the PZT thin film undergoes elongation under positive voltage excitation and contraction under negative voltage excitation. When a sinusoidal signal at the ultrasonic driving frequency is applied to the microrobot, its actuation behavior is represented on the right side of Fig. 2a. In addition to the elongation and contraction of the PZT thin film, the ultrasonic frequency actuation induces a wave-like mode in the body of the robot, resembling the multi-legged motion of a centipede. This microscopic deformation of the piezoelectric material is then transformed into macroscopic motion through mechanical resonance and frictional coupling with the inner wall of the pipe, enabling controlled high-speed movement in confined tubular environments.

To determine the optimal driving frequency, it is essential to identify the resonant frequency of the designed microrobot. FEA was performed using COMSOL Multiphysics. The microrobot was simplified into a three-layer thin plate model, representing the PZT layer, the conductive silver epoxy resin layer, and the flexible PET substrate from top to bottom (Fig. S2). After constructing the model and generating the mesh, a frequency domain study was performed in COMSOL Multiphysics. The sweep was set from 40 to 80 kHz with a 1 kHz step size to reduce computation time. In the Results section, a global calculation parameter of the impedance magnitude was specified to obtain the impedance at each frequency point. Plotting these data produces the impedance spectrum, where the frequency corresponding to the minimum impedance identifies the resonance frequency, with the material parameters used in the simulation summarized in Table S1. The simulated impedance characteristic curve is shown as the blue curve in Fig. 2b, indicating that the resonant frequency of the designed microrobot is 60 kHz. To validate this result, the impedance characteristics of the fabricated microrobot were tested using an impedance analyzer. The experimental results are shown as the red curve in Fig. 2b, revealing a discrepancy of 2.45 kHz (4.26%) between the simulated and measured results. This deviation is primarily attributed to minor fabrication errors and the absence of damping considerations in the simulation analysis. Additionally, the vibration characteristics of the robot were tested using a laser Doppler vibrometer (Fig. S3). The vibration velocity response spectrum as a function of frequency is shown in Fig. 2c, with the inset depicting the vibration mode at the maximum vibration velocity. The results indicate that the robot achieves its maximum vibration velocity at 57.41 kHz, closely aligning with the experimentally measured resonant frequency of 57.55 kHz. The slight discrepancy is mainly due to differences in the working planes under the two testing conditions. These findings demonstrate the excellent stability of the fabricated robot and confirm that it achieves maximum vibration velocity near the resonant frequency, offering valuable guidance for realizing high-speed motion in narrow pipe environments.

Performance tests were conducted on the fabricated microrobot, using a circular glass pipe with an inner diameter of 21 mm as the test environment. An ultrasonic driving signal was generated by a waveform generator, amplified with a voltage amplifier, and supplied to the robot through external silver wires. The microrobot was placed inside the pipe for motion performance testing. A schematic of the experimental setup is shown in Fig. S4. An oscilloscope monitored the output voltage and current from the voltage amplifier, while an optical camera recorded the motion of the robot. The relationship between the speed of the robot in the glass pipe and the driving frequency at a fixed driving voltage of 50 V_P-P_ is shown in Fig. 2d. It was observed that as the driving frequency increased, the motion direction of the robot within the pipe changed. Specifically, within the frequency range of 45-49 kHz, the motion direction of the robot reversed from forward to backward, while beyond 50 kHz, it returned to forward motion. For backward motion, the maximum speed was 31.2 cm s^−1^ at 48 kHz (averaged over three tests). For forward motion, the maximum speed was 68.8 cm s^−1^ at 55 kHz (averaged over three tests). To pinpoint the frequency corresponding to the maximum speed, a finer test was conducted within the 54-55 kHz range using a step size of 0.1 kHz. The results are shown in Fig. 2e, indicating that the robot achieved its highest speed of 81 cm s^−1^ at 54.8 kHz, exceeding that of the fastest piezoelectric microrobots. To investigate the effect of driving voltage amplitude on the speed of the robot, the driving frequency was fixed at 54.8 kHz, and the speed of the robot was tested under different driving voltages. As shown in Fig. 2f, the speed of the robot increased approximately linearly with the driving voltage. This behavior is consistent with expectations, as higher driving voltages increase vibration amplitude of the robot, leading to faster motion [44–46].

The test results shown in Fig. 2d demonstrate that the robot exhibits different motion directions within the pipeline at different frequency ranges. This capability is crucial for performing inspection tasks in pipelines. If the robot were limited to unidirectional motion during inspection, it would significantly reduce inspection efficiency, and under certain conditions, might render some tasks infeasible. To further explain the motion mechanism of the microrobot, FEA was performed using COMSOL Multiphysics to conduct modal analysis of the robot at characteristic frequencies [44]. Figure 2g illustrates the forward motion mode at the resonant frequency of 59.821 kHz (Movie S1). In this state, the body exhibits a forward–backward oscillatory motion, characterized by a complex high-order vibration mode. At the contact points with the inner wall of the glass tube, modal coupling induces elliptical motion trajectories [47, 48], generating propulsion by overcoming friction in the forward direction and enabling movement. For backward motion, simulations reveal a backward motion mode at 47.268 kHz (Fig. 2h, Movie S1). The vibrational mode changes with different ultrasonic resonant frequencies. In this mode, the body exhibits an asymmetric nodal distribution during movement. At the contact points with the inner wall of the glass tube, an elliptical motion trajectory opposite to that of forward motion is formed, reducing friction in the backward direction and facilitating movement in reverse.

### Motion Testing and Analysis of the Microrobot

**Fig. 3:**
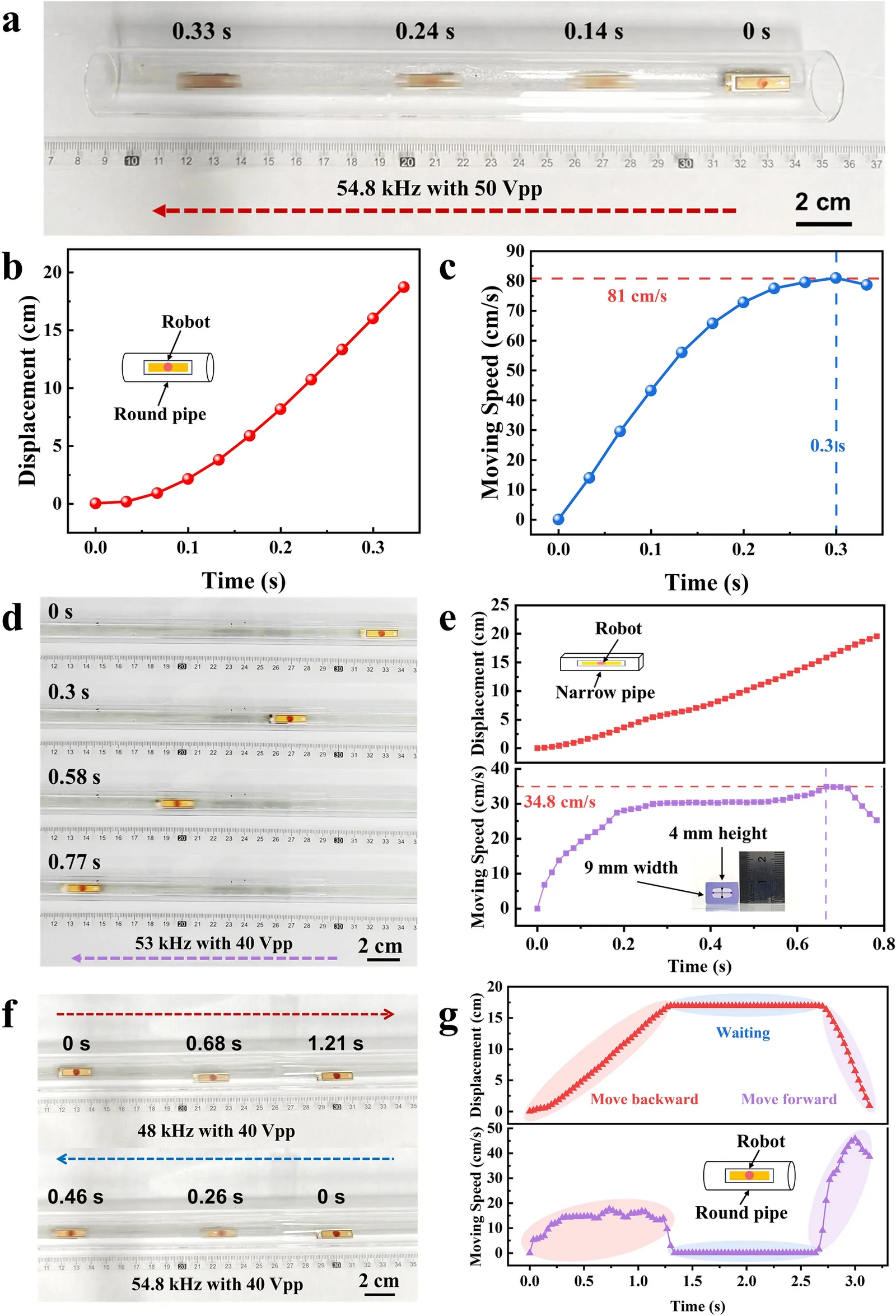
Motion demonstration and analysis of the microrobot in pipes of various shapes. **a** Motion demonstration of the microrobot in a circular glass pipe with an inner diameter of 21 mm. **b-c** Real-time displacement and velocity profiles of the microrobot during motion, showing the fastest speed of the robot is 81 cm s^−1^. **d** Motion demonstration of the microrobot in a narrow square pipe with an inner diameter of 9 mm and a height of 4 mm. **e** Real-time displacement and velocity profiles of the microrobot during motion, showing the fastest speed of the robot is 34.3 cm s^−1^. **f** Bidirectional motion demonstration of the microrobot in a circular glass pipe. **g** Real-time displacement and velocity profiles of the microrobot during bidirectional motion, indicating that the robot has a faster movement speed while moving forward

We conducted motion tests of the microrobot in a circular glass pipe with an inner diameter of 21 mm, setting the driving frequency to 54.8 kHz and the driving voltage to 50 V_P-P_. The motion of the robot inside the pipe was recorded using an optical camera (Fig. 3a). Further analysis focused on the real-time displacement and velocity of the robot, as shown in Fig. 3b, c. The results revealed that the robot reached a maximum speed of 81 cm s^−1^ within 0.3 s, demonstrating its exceptional high-speed motion capability within pipes. Subsequently, the pipe shape was changed to a square acrylic pipe with an inner diameter of 18 mm. Performance tests were conducted at a driving frequency of 48 kHz and a driving voltage of 50 V_P-P_ (Fig. S5). The robot achieved a maximum speed of 55.5 cm s^−1^ within 0.25 s. To further evaluate the performance of the robot in millimeter-scale narrow pipes, we tested its motion in a square pipe with an inner diameter of 9 mm and a height of 4 mm (Fig. 3d, e). Even in this narrow pipe with a height of only 4 mm, the robot maintained high-speed operation, achieving a maximum speed of 34.3 cm s^−1^. The speed reduction is primarily attributed to changes of the pipeline environment. In circular glass pipes, the contact area between the robot and the inner wall is mainly concentrated on its left and right sides. In contrast, in square pipes, the contact area encompasses the entire body of the robot, resulting in larger friction force that restricts its movement speed. To further assess the maximum travel distance of the robot in tethered mode, we performed experiments in a circular glass tube with an inner diameter of 21 mm and a length of 40 cm (Fig. S6); these dimensions were chosen to stay within the 50 cm length of the electrode leads. The results demonstrate that the robot can traverse the entire 40 cm micro-pipe stably and without difficulty. Using a dynamical difference method, we estimated that the friction force generated by the electrode wires during the motion of the robot is approximately 0.1 mN (Fig. S7).

Finally, the bidirectional motion capability of the robot was tested by placing it in a circular glass pipe with an inner diameter of 21 mm. The driving frequency was initially set to 48 kHz, then turned off, and subsequently set to 54.8 kHz with a driving voltage of 40 V_P-P_. The results, shown in Fig. 3f, g, indicate that the speed of the robot during backward motion was lower than during forward motion, consistent with our previous findings (Fig. 2d). The speed difference in forward and backward motion is mainly attributable to variations in the driving frequency and the corresponding vibration modes. During forward motion, the excitation frequency is closer to the resonant frequency, yielding larger vibration amplitudes and greater thrust. In addition, different driving frequencies excite distinct vibration modes, altering the contact coupling pattern between the robot and the pipe wall and further affecting the running speed. These results demonstrate that the robot can achieve directional switching within the pipe by simply adjusting the driving frequency, offering significant advantages for practical pipe inspection tasks and enhancing inspection efficiency. Movie S2 demonstrates the motion of the robot in different pipes, including its bidirectional motion capabilities.

### Demonstration of Driving Performance, Climbing and Load-Bearing Capabilities

**Fig. 4:**
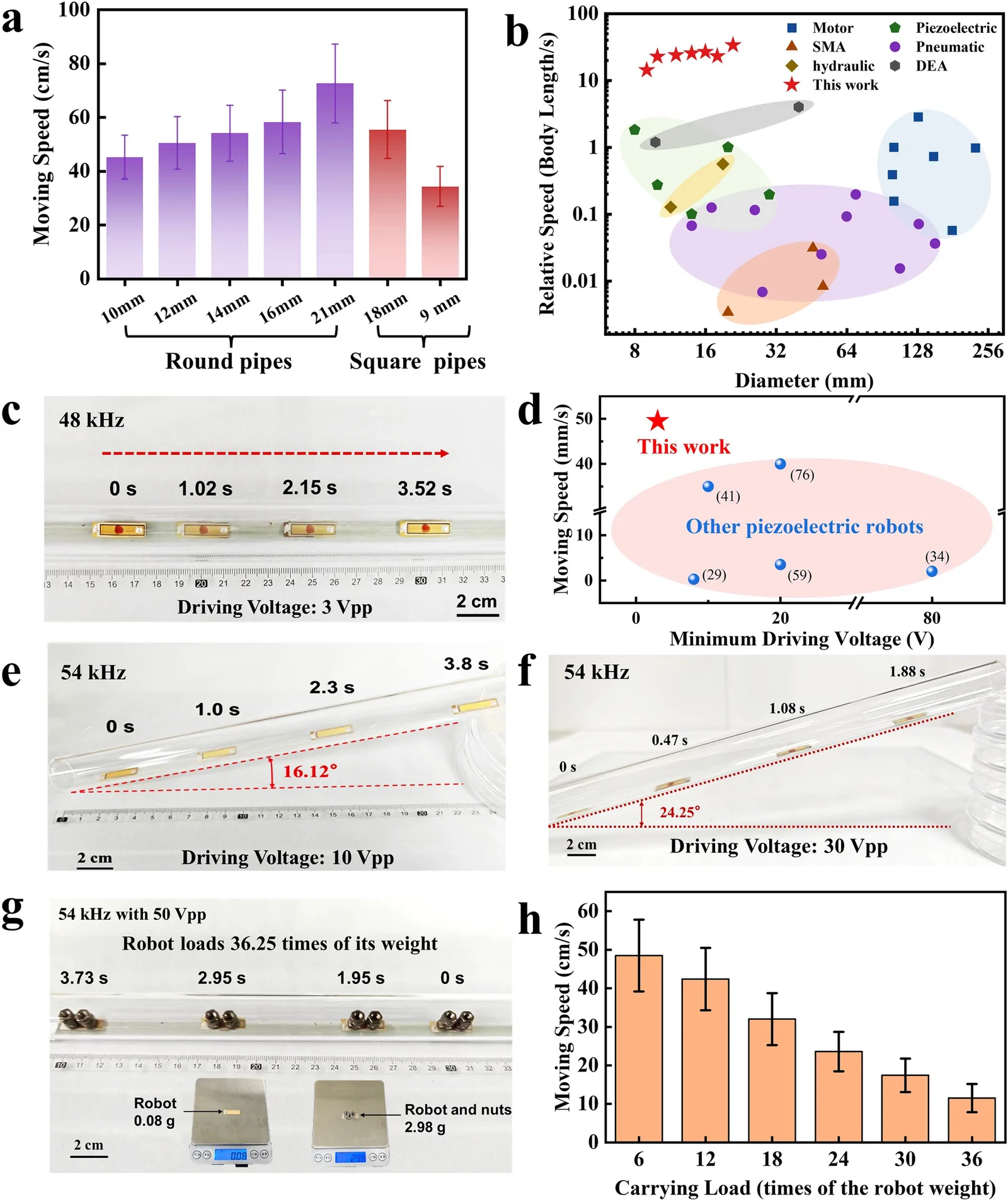
Comparison of driving performance and demonstration of climbing and load-bearing capabilities. **a** Comparison of the motion speed of the microrobot in pipes with varying inner diameters. **b** Comparison of working pipe dimensions and motion speeds of pipeline robots employing different driving principles. **c** Motion demonstration of the microrobot at a driving voltage of 3 V_P-P_. **d** Comparison of the minimum driving voltages and corresponding motion speeds of various piezoelectric microrobots. **e** Demonstration of the microrobot climbing a 16.12° slope at a driving voltage of 10 V_P-P_. **f** Demonstration of the microrobot rapidly climbing a 24.25° slope at a driving voltage of 30 V_P-P_. **g** Motion demonstration of the microrobot (80 mg loaded with nuts 2.9 g) over 36 times its weight; **h** Motion speed comparison of the microrobot loaded with different weights. The error bars represent the standard deviation of three measurements

We evaluated the motion of the robot in pipes with variable inner diameters, with the results presented in Fig. 4a and Movie S2. In Fig. 4a, the purple data correspond to tests conducted in circular glass pipes, while the red data represent results from square acrylic pipes. The experimental findings indicate that the motion speed of the robot increases as the pipe diameter enlarges, because the friction between the robot and the wall of pipe increases as the diameter is reduced. To provide a broader perspective, we created a comparison chart (Fig. 4b) illustrating the applicable pipe diameters and motion speeds of robots employing various driving principles, including electromagnetic motors [49–55], piezoelectric actuators [56–59], shape memory alloy (SMA) actuators [60–62], pneumatic systems [63–72], liquid-driven mechanisms [73, 74], and dielectric elastomer actuators [4, 75]. Our proposed piezoelectric-driven ultrasonic microrobot demonstrates high-speed motion in mm-to-cm scale pipes (9-27 mm), with relative speeds far exceeding those achieved by robots based on other driving principles.

We conducted experiments to determine the minimum driving voltage of the fabricated robot and found that it could achieve initial motion at a driving voltage as low as 3 V_P-P_ (Fig. 4c and Movie S3). This is primarily attributed to the advantages of the piezoelectric composite film microstructure and ultrasonic frequency actuation. The driving voltage is two orders of magnitude lower than that required by DEAs [17], underscoring the excellent safety performance of the robot in terms of power supply. To further contextualize this achievement, we compared the minimum driving voltage of our robot with that of other piezoelectric robots and plotted a comparison chart illustrating the minimum driving voltage alongside the corresponding motion speeds (Fig. 4d) [29, 34, 41, 59, 76]. The results demonstrate that our robot operates at a significantly low driving voltage while achieving high motion speeds, showcasing remarkable advantages in energy efficiency and operational performance.

We measured the RMS current of the robot at driving voltages of 3 V_P-P_ and 30 V_P-P_ with a high-precision digital multimeter (DMM6500) (Fig. S8). Combining these currents with the corresponding input voltages yields input powers of 4.73 mW at 3 V_P-P_ and 0.515 W at 30 V_P-P_. To evaluate long-term operational endurance, we monitored surface temperature with an infrared thermal camera (FOTRIC 228 s) before and after one minute of continuous locomotion (Fig. S9). At 10 V_P-P_ the robot exhibited virtually no heating after one minute. Notably, even at 20 V_P-P_ the surface temperature rose by only 7.4-35.7 °C, corresponding to a heating rate of ~ 0.12 °C s^−1^, which is markedly lower than that of conventional piezo-ultrasonic devices.

We further evaluated the climbing and load-bearing capabilities of the robot. The results demonstrate that the robot could stably climb a 16.12° slope with an average speed of 5.34 cm s^−1^ at a driving voltage of 10 V_P-P_ and rapidly climb a 24.25° slope with an average speed of 13.14 cm s^−1^ at a driving voltage of 30 V_P-P_ (Fig. 4e, f, Movie S4). Additionally, tests conducted in pipes with varying diameters (Fig. S10, Movie S4) revealed that the robot exhibits superior climbing performance when navigating narrow pipes of different dimensions. For load-bearing tests, the load weight was controlled by attaching varying numbers of nuts to the robot. At a driving voltage of 50 V_P-P_, the robot was capable of carrying a load exceeding 36 times its weight while maintaining a relatively high speed of 11.5 cm s^−1^ (average of three measurements) (Fig. 4g, Movie S4). The relationship between the speed of the robot and its load (expressed as multiples of its weight) is presented in Fig. 4h. These results highlight the exceptional robustness of the robot and outstanding performance in load-bearing tasks.

### Motion Experiments of the Microrobot in Complex Environments

**Fig. 5:**
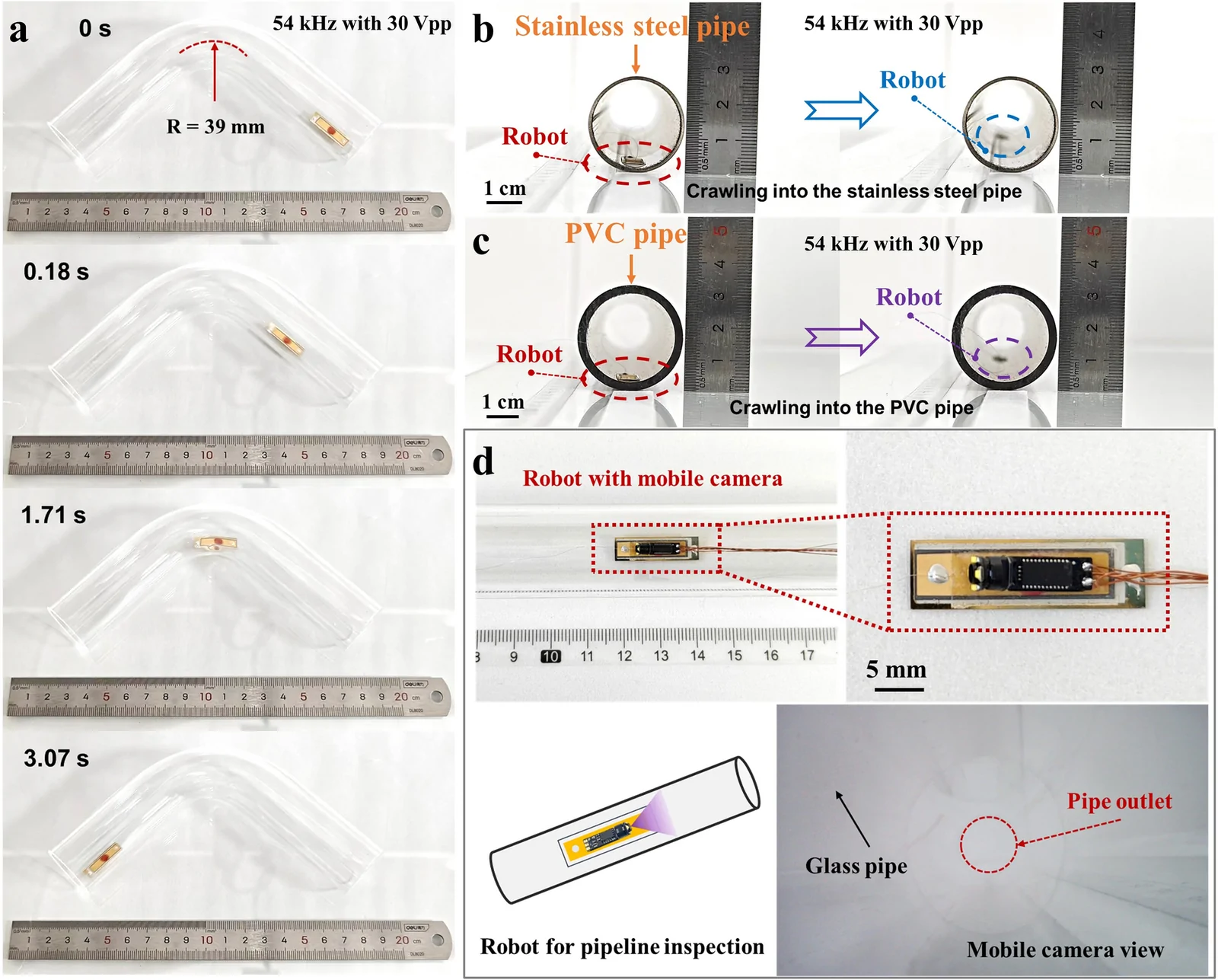
Motion demonstration of the microrobot in curved pipes, pipes made of different materials, and its ability to perform inspection tasks with a mounted micro camera. **a** Demonstration of the microrobot navigating in an L-shaped pipe with a turning radius of 39 mm. **b** Microrobot climbed into a stainless steel pipe. **c** The microrobot climbed into a PVC pipe. **d** Robot equipped with a micro-endoscope camera and an image captured during the inspection of the environment inside a transparent glass pipe

We tested the motion of the microrobot in a curved L-shaped pipe with a turning radius of 39 mm. The robot successfully navigated through the L-shaped pipe with an average speed of 4.06 cm s^−1^ (Fig. 5a, Movie S5). The results demonstrate that the robot maintains excellent performance in curved pipe environments, broadening its application potential in practical scenarios. We also evaluated the bidirectional motion capability of the robot in pipes made of different materials, recording its motion using an optical camera (Fig. 5b, c and S11, Movie S6). The inner diameters of the stainless steel and polyvinyl chloride (PVC) pipes were 27 and 32 mm, respectively, the robot was driven at a fixed voltage of 30 V_P-P_. The results indicate that the robot adapts effectively to various pipe surface conditions. However, the friction coefficient of the pipe material impacts the speed of the robot, suggesting that the driving frequency may need to be adjusted to achieve optimal performance under different conditions. Additionally, the ability of the microrobot to move over water was demonstrated. Unlike solid pipe surfaces, water presents a completely different medium, with motion influenced by surface tension and buoyancy. To overcome water surface tension, the driving frequency was adjusted to 1 kHz, enabling the robot to generate larger vibration amplitudes and achieve motion (Fig. S12, Movie S7). It was further shown that our robots have excellent encapsulation properties.

To demonstrate the practical application of the robot in pipe inspection, a micro-endoscope camera (EZ-EN35S-S from Shenzhen Ezon Electronics Co., Ltd; 200 mg) was mounted on the robot and tested in a circular glass pipe with an inner diameter of 21 mm. The camera was connected to a mobile computer via an external wire harness for real-time image recording inside the pipe (Fig. 5d, Movie S8). Notably, when the robot moved rapidly within pipes, the limited precision and stability of the miniature camera caused severe image jitter. In practical applications, controlling the motion speed of the robot is necessary to obtain stable images. For long-distance inspection in narrow pipes, implementing anti-shake mechanisms and utilizing wireless high-speed miniature cameras would significantly enhance inspection performance.

## Conclusions

In this work, we demonstrated an ultrasonic microrobot with ultra-fast motion speed and low-voltage driving capabilities, successfully applied to the inspection of narrow pipes with a broad diameter range (9-27 mm). The robot utilized a thinned PZT film as the driving unit, integrated with a flexible PET substrate to form a piezoelectric composite film microstructure, and encapsulated with a parylene-C film. By simply adjusting the ultrasonic driving frequency and voltage, the robot demonstrated bidirectional high-speed motion capabilities across various narrow pipes. The robot achieved a motion speed of 81 cm s^−1^, surpassing the fastest piezoelectric microrobots. Moreover, the robot exhibited initial motion at a remarkably low driving voltage of 3 V_P-P_, suggesting significant potential for future integration of onboard circuits to enable untethered operation. The robot also demonstrated exceptional robustness in climbing and load-bearing performance. Furthermore, it showed versatility by achieving rapid motion in curved pipes, pipes made of different materials, and even over water. Compared to pipeline robots based on other operating principles, the robot developed in this work offered greater adaptability to pipe diameters and achieved superior high-speed bidirectional navigation, particularly for millimeter-scale pipe inspections. For practical applications, we conducted a preliminary demonstration of the robot navigating in glass pipes and recording images, showcasing its potential for pipe inspection tasks in complex systems.

Although promising, there are still several aspects of our robot that need to be further optimized. Enhancing the structural design or incorporating driving units with higher power density will be effective to improve the driving performance of the robot. Furthermore, the robot relies on external electrode wires for power, thereby restricting its capability for long-distance pipe inspection tasks. Future iterations of the robot will be equipped with miniature onboard power supply circuits, communication modules, and micro-batteries, enabling remote-controlled autonomous operation and expanding its practical applicability [77].

## Supplementary Information

Below is the link to the electronic supplementary material.

Supplementary file1 (MP4 387 KB)

Supplementary file2 (MP4 1414 KB)

Supplementary file3 (MP4 299 KB)

Supplementary file4 (MP4 1196 KB)

Supplementary file5 (MP4 311 KB)

Supplementary file6 (MP4 1205 KB)

Supplementary file7 (MP4 319 KB)

Supplementary file8 (MP4 1559 KB)

Supplementary file9 (DOCX 8482 KB)
