# Supplementary material for: An Ultrasonic Microrobot Enabling Ultrafast Bidirectional Navigation in Confined Tubular Environments
Source: Nanomicro Lett. 2025 Aug 25;18:43. doi: 10.1007/s40820-025-01894-y (PMC12378850; doi:10.1007/s40820-025-01894-y)
Supplement: Supplementary file 9 — Supplementary file9 (DOCX 8482 KB) [file 40820_2025_1894_MOESM9_ESM.docx]

Supporting Information for

**An Ultrasonic Microrobot Enabling Ultrafast** **Bidirectional Navigation in Confined Tubular Environments**

Meng Cui^1,2^, Liyun Zhen^1,2^, Xingyu Bai^1,2^, Lihan Yu^1,2^, Xuhao Chen^1,2^, Jingquan Liu^1^, Qingkun Liu^1^, Bin Yang^1*^

^1^National Key Laboratory of Advanced Micro and Nano Manufacture Technology, Shanghai Jiao Tong University, Shanghai 200240, P. R. China

^2^Department of Micro/Nano Electronics, School of Electronic Information and Electrical Engineering, Shanghai Jiao Tong University, Shanghai 200240, P. R. China

^*^ Correspondence author. E-mail: [binyang@sjtu.edu.cn](mailto:binyang@sjtu.edu.cn) (Bin Yang)

**Supplementary Figures**


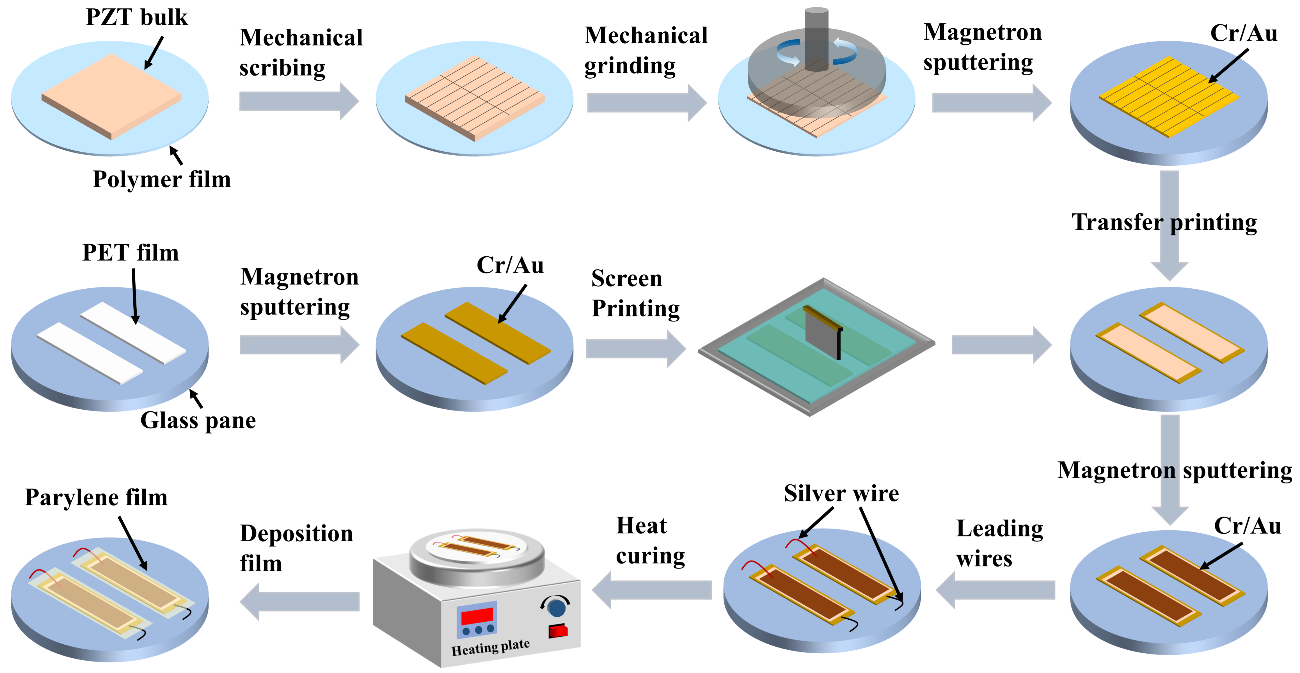


**Fig. S1** The fabrication process of the ultrasound microrobot

The fabrication process of the robot involves four primary steps as follows: (1) mechanical thinning and patterning of the PZT substrate, (2) sputtering Cr/Au electrodes on the surface of the PET and PZT, (3) transfer bonding and Cr/Au electrode deposition on the top surface of the PZT, and (4) electrode wiring and parylene-C film encapsulation. In the first step, the PZT substrate (300 μm thick) is mechanically diced to obtain a patterned thin film, and subsequently thinned via mechanical grinding to achieve a uniform thickness of 70 μm. In the second step, a Cr/Au electrode layer (20/200 nm) is sputtered onto the surface of the PZT thin film via magnetron sputtering, while an identical Cr/Au electrode layer is sputtered onto the patterned PET surface. In the third step, a conductive silver epoxy resin layer is printed onto the PET surface using a screen-printing process, and the PZT thin film is transferred onto the PET substrate, followed by curing at 85°C for 3 hours. The screen-printing technique ensures the uniformity and consistency of the conductive silver epoxy resin, providing reliable electrical connections between the PET and PZT layers. A Cr/Au electrode is then sputtered onto the top surface of the PZT thin film. In the fourth step, two silver wires (50 μm in diameter) are attached to the top surfaces of the PET and PZT using conductive silver epoxy resin to serve as signal drive wires for the micro-robot. The robot is then cured at 85°C for 3 hours to secure the silver wire connections. Finally, a 10 μm-thick parylene-C film is deposited on the surface of the robot as an encapsulation layer, protecting the micro-robot during operation. The overall thickness of the fabricated micro-robot is approximately 210 μm.


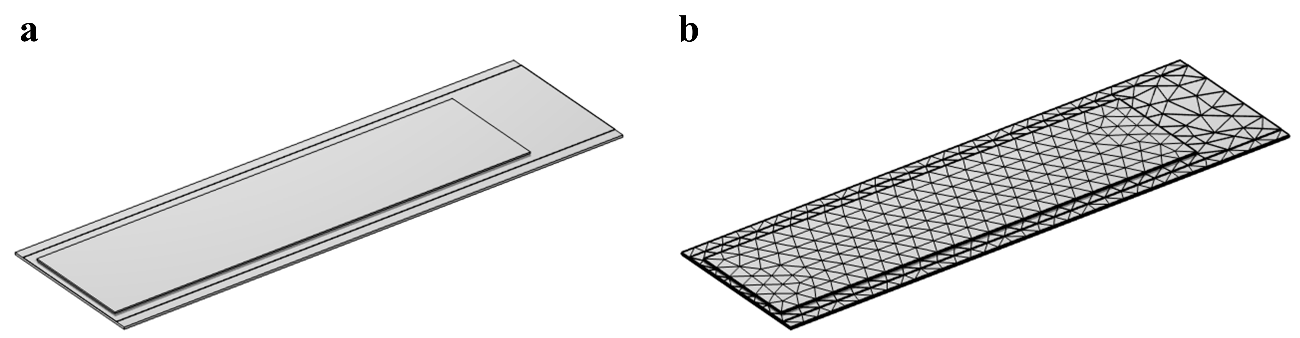


**Fig. S2** Simplified model of the robot built in COMSOL Multiphysics.  **(a)** Simplified model of the robot as a three-layer thin plate. **(b)** Meshing of the simplified model


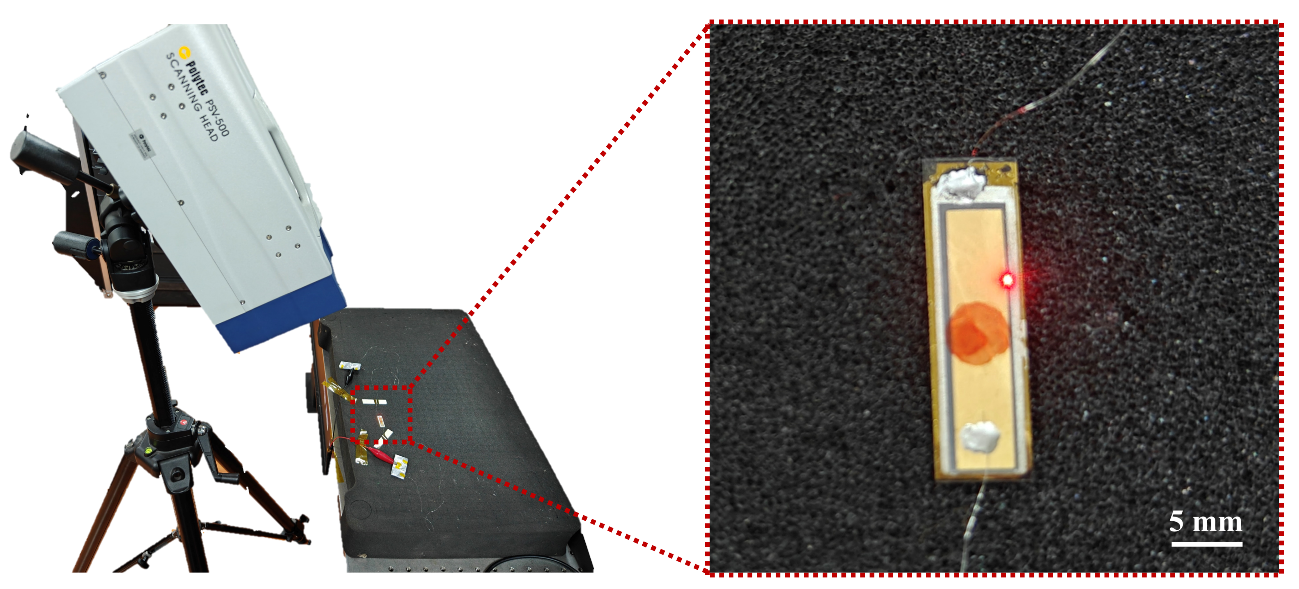


**Fig. S3.** Vibration modes of the microrobot were tested using a laser Doppler vibrometer


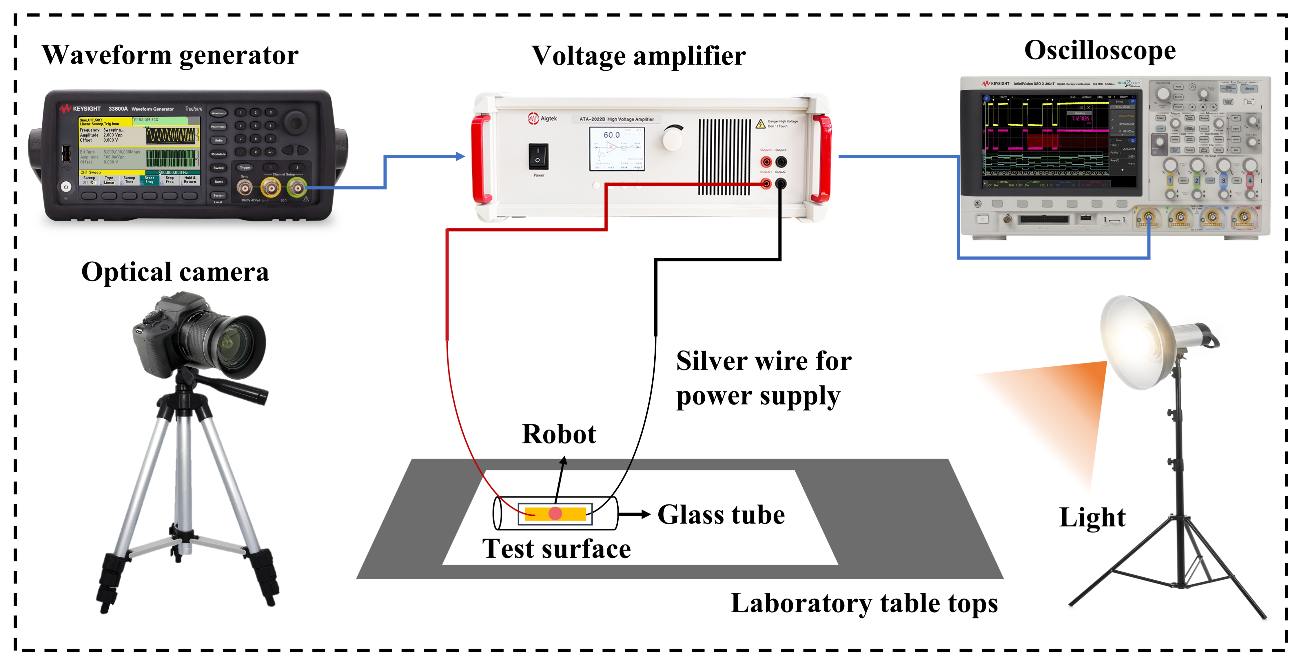


**Fig. S4** Schematic diagram of the test setup for the microrobot


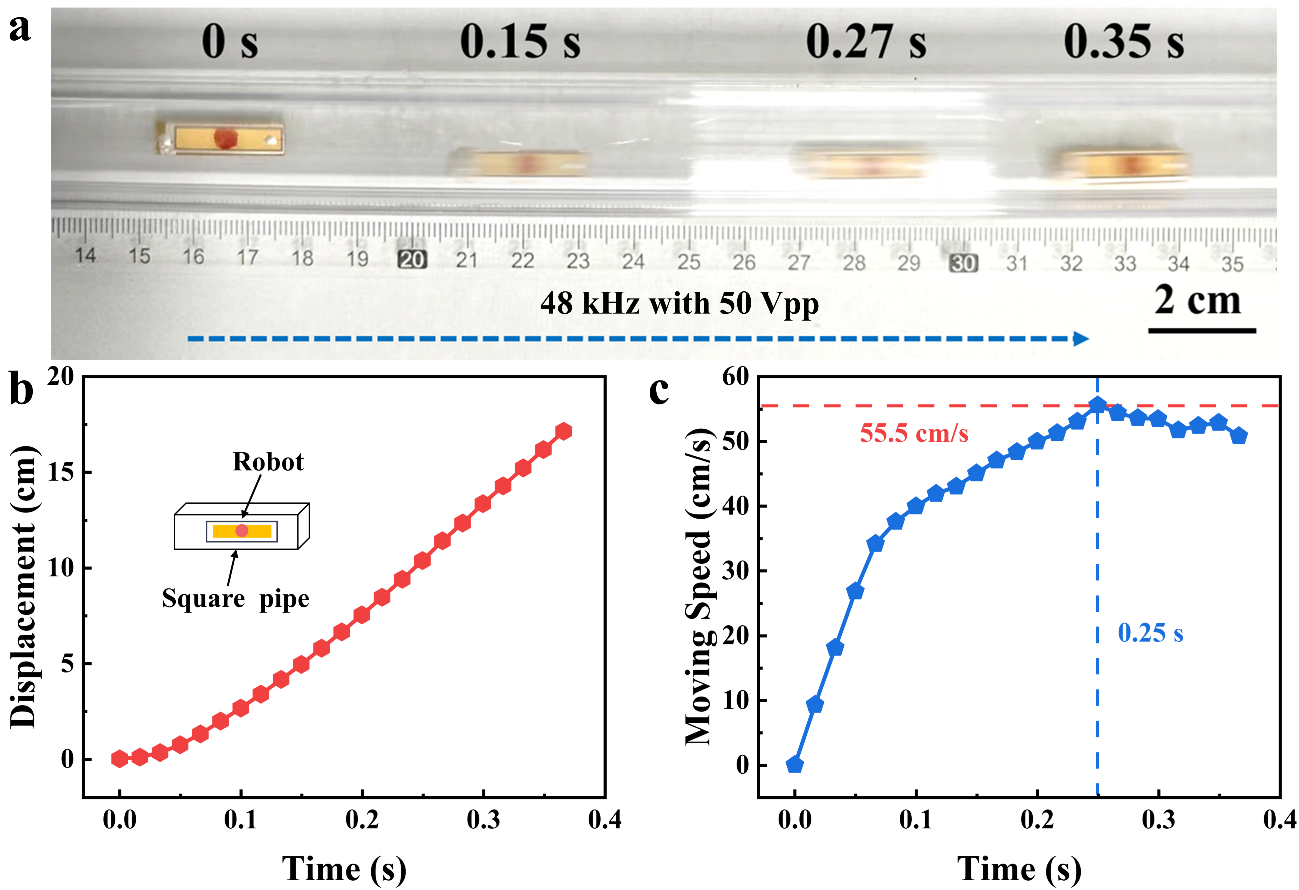


**Fig. S5** Motion demonstration of the microrobot in the square pipe with an inner diameter of 18mm


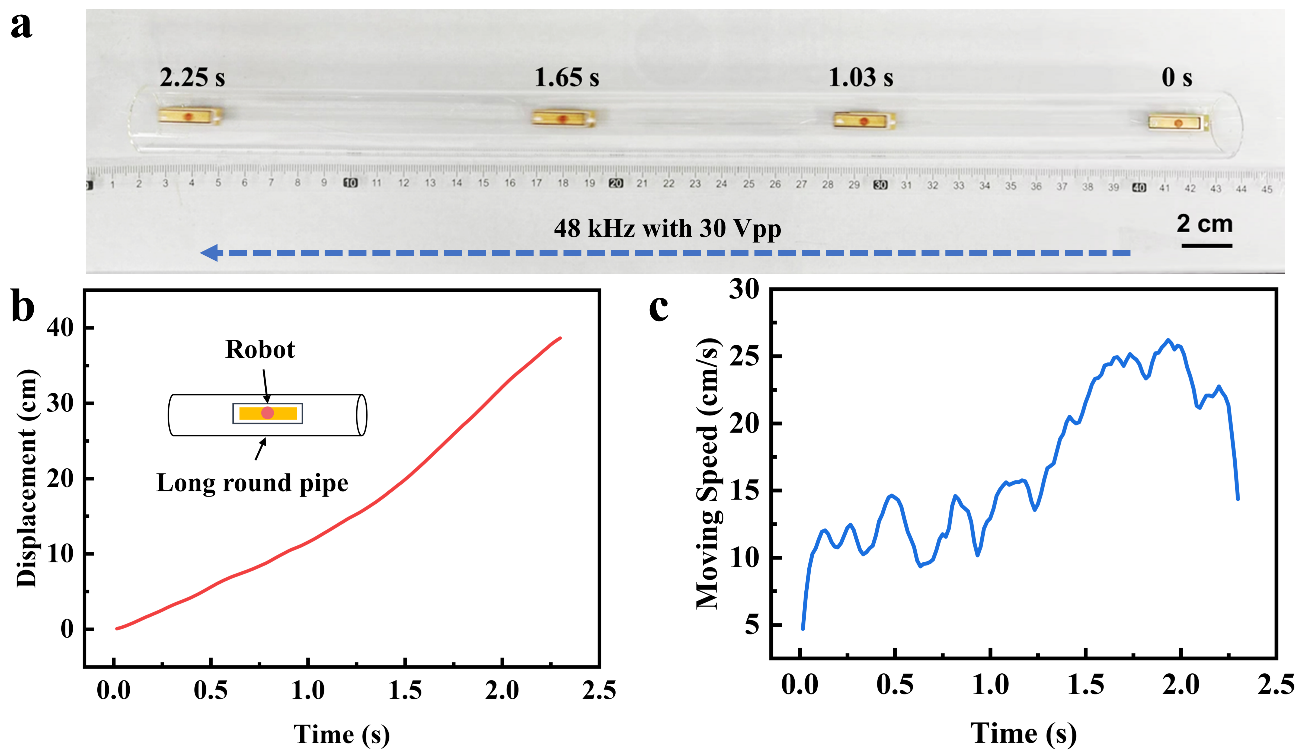


**Fig. S6** Motion demonstration of the microrobot in the circular pipe with a length of 40 cm and an inner diameter of 18mm


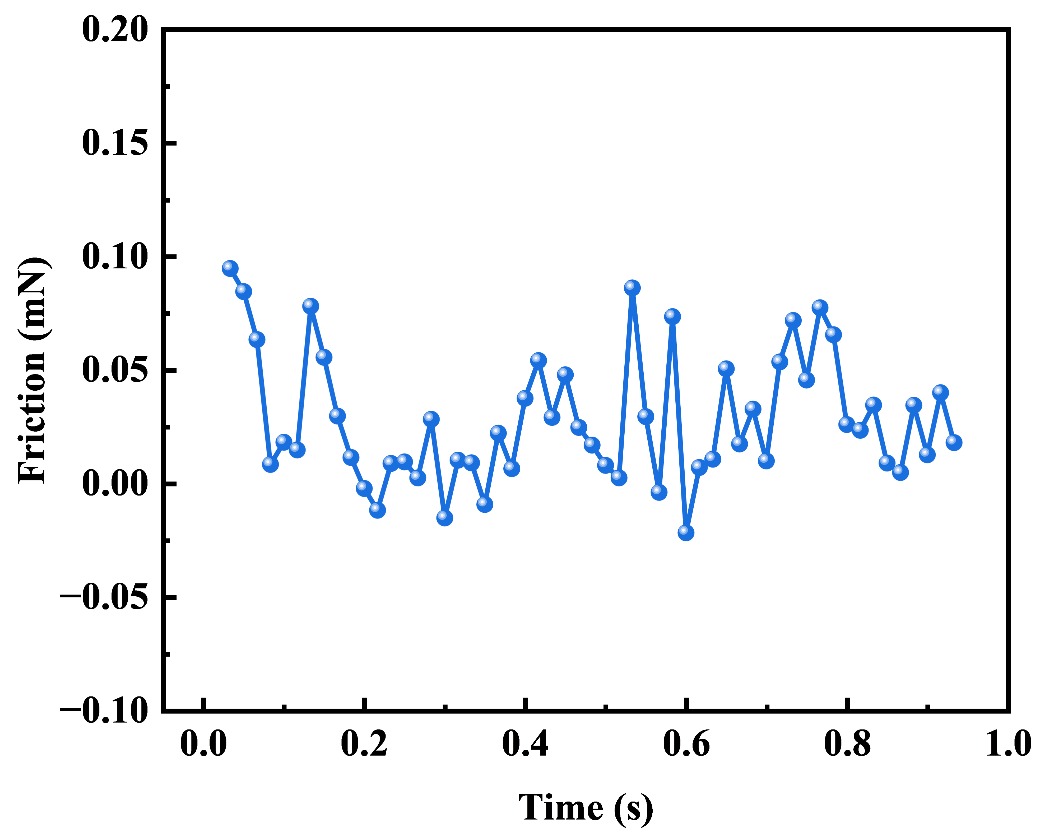


**Fig. S7** The estimated friction force generated by the electrode wires when they are pulled along by the motion of the robot

We employed a dynamical-difference method to obtain an approximate estimate of the additional frictional resistance introduced by the electrode leads when the robot operates in tethered mode.

**Experimental rationale and procedure**

**(1) Governing equations**

**Untethered case:** When the robot is not subjected to cable friction, Newton’s second law gives

$$F_{drive}=ma_{0}$$

Where $F_{drive}$ is the propulsive force, m is the mass of the robot, and $a_{0}(t)$ is its time-dependent acceleration (the robot does not move at constant velocity).

**Tethered case:** When cable friction is present,

$$F_{drive}=ma_{1}+F_{cable}$$

so that, after neglecting minor effects such as fluid drag,

$$F_{cable}=m(a_{0}-a_{1})$$

With m=80 mg, knowledge of $a_{0}(t)$ and $a_{1}(t)$ suffices to estimate $F_{cable}$.

**(2) Acceleration without cable friction.**

The electrode leads were suspended on a rigid frame so that they remained clear of the track; the robot’s motion was recorded with an optical camera, and post-processing of the video yielded the instantaneous acceleration $a_{0}(t)$.

**(3) Acceleration with cable friction.**

The leads were then laid flat on the track, allowing friction to develop between the cable and the surface during locomotion. A second video recording provided the corresponding acceleration profile $a_{1}(t)$.

**(4) Friction-load calculation.**

Point-by-point subtraction of the two acceleration curves, followed by multiplication by the robot’s mass, produced the estimated cable friction force. As shown in the accompanying figure, the resulting value is approximately 0.1 mN.


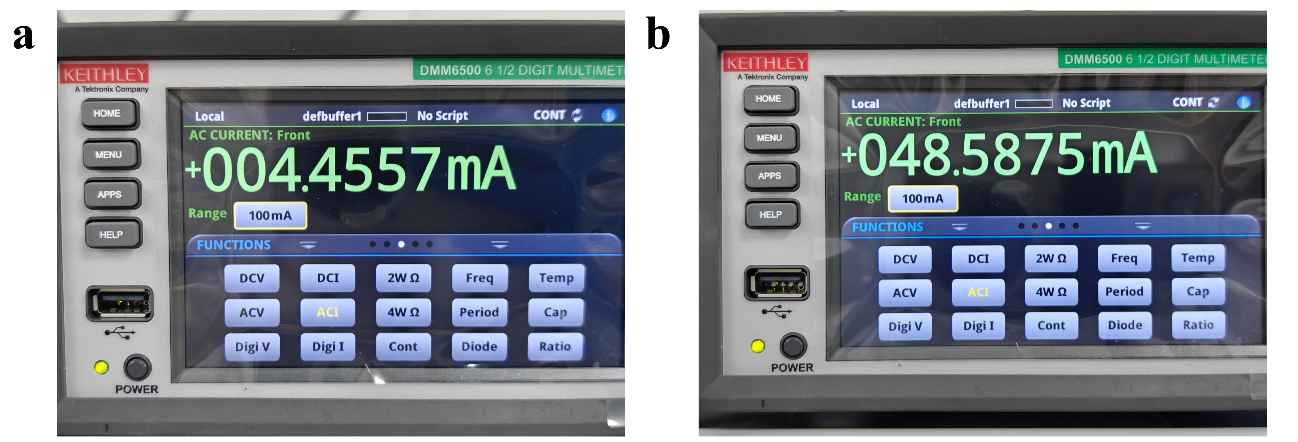


**Fig. S8** Testing the working current of the microrobot using high-precision digital multimeter (DMM6500). **(a)** Driving voltage of 3 V_P-P_, **(b)** Driving voltage of 30 V_P-P_


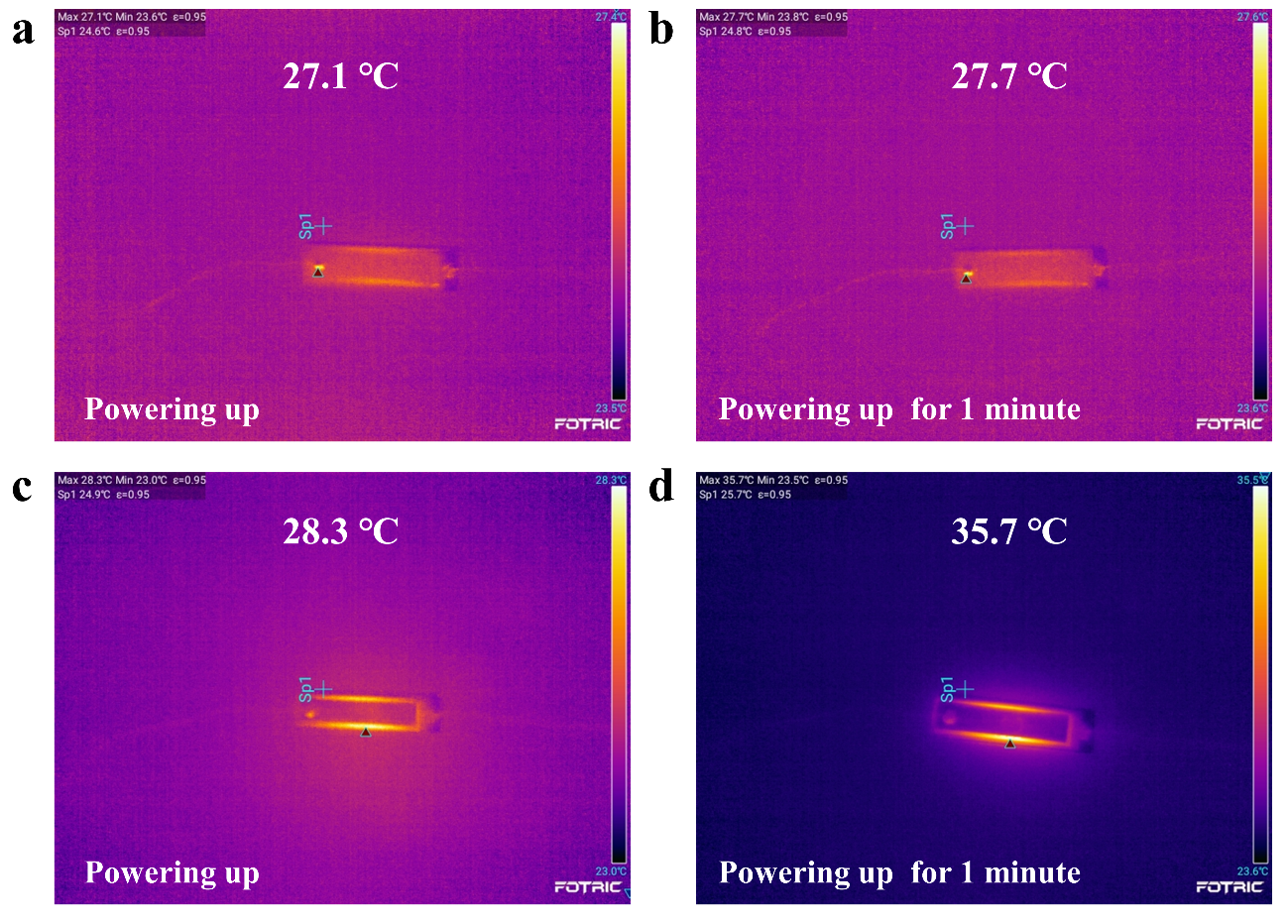


**Fig. S9** The temperature change of the robot before and after 1 minute of motion was monitored using infrared thermal imager (FOTRIC 228s). **(a-b)** Driving voltage of 3 V_P-P_, **(c-d)** Driving voltage of 20 V_P-P_


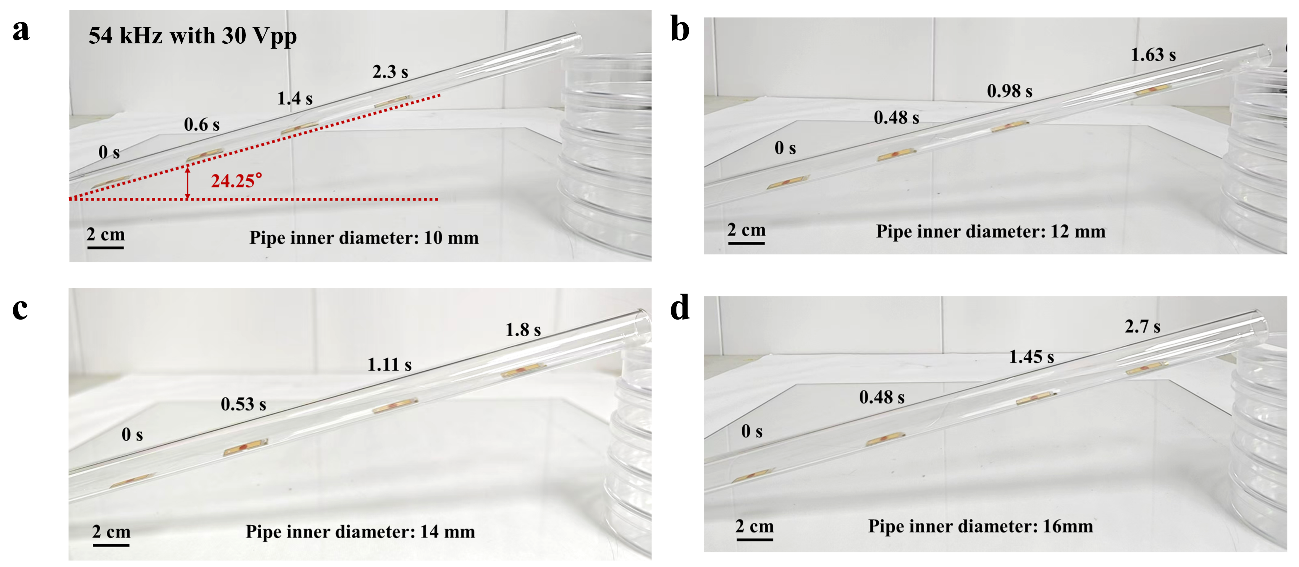


**Fig. S10** The climbing ability of the robot in pipes with different inner diameters


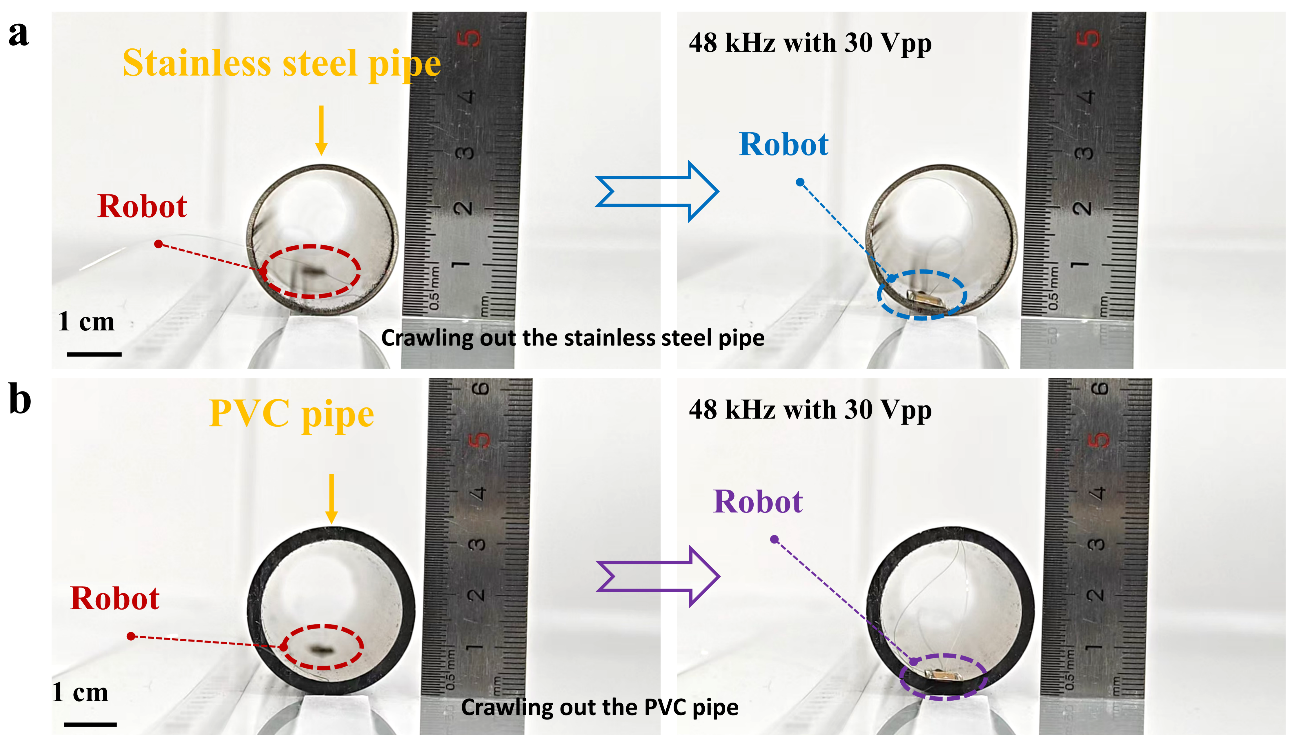


**Fig. S11** The robot crawled out the stainless steel and PVC pipes


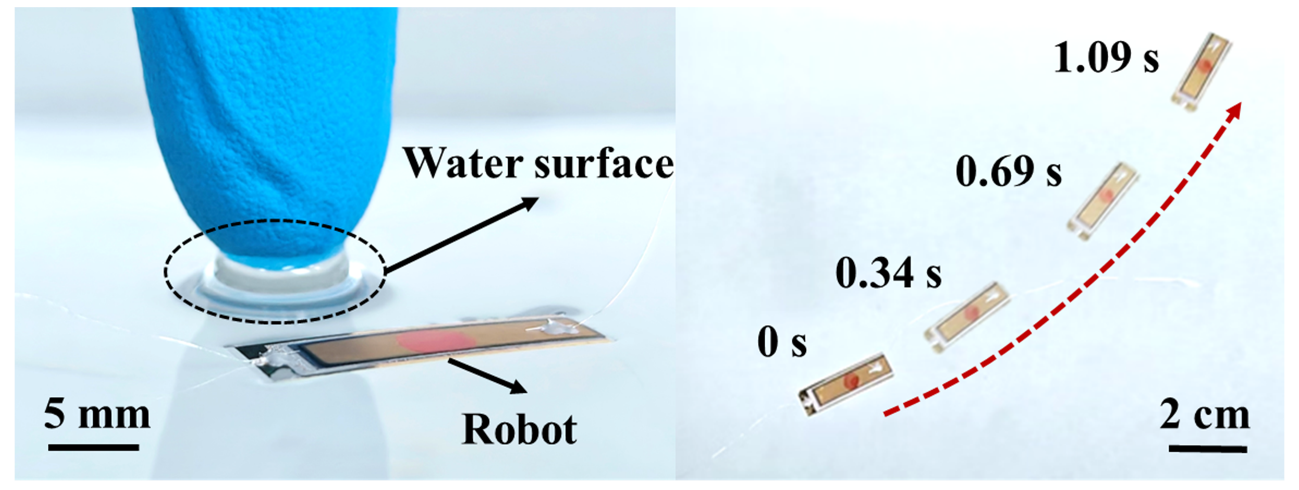


**Fig. S12** Motion demonstration of the robot over water

**Table S1** Material properties of FEA simulation

| **Material** | **PZT** | **Conductive epoxy** | **PET** |
| --- | --- | --- | --- |
| Density (kg/m^3^) | 7500 | 3750 | 1380 |
| Young’s modules (GPa) | --- | 45 | 3 |
| Poisson’s ratio | --- | 0.25 | 0.38 |
| Elasticity matrix (GPa) | $\left[\begin{matrix} \mathbf{127.21} & \mathbf{80.21} & \mathbf{84.67} & \mathbf{0} & \mathbf{0} & \mathbf{0} \\ \mathbf{80.21} & \mathbf{127.21} & \mathbf{84.67} & \mathbf{0} & \mathbf{0} & \mathbf{0} \\ \mathbf{84.67} & \mathbf{84.67} & \mathbf{117.44} & \mathbf{0} & \mathbf{0} & \mathbf{0} \\ \mathbf{0} & \mathbf{0} & \mathbf{0} & \mathbf{22.99} & \mathbf{0} & \mathbf{0} \\ \mathbf{0} & \mathbf{0} & \mathbf{0} & \mathbf{0} & \mathbf{22.99} & \mathbf{0} \\ \mathbf{0} & \mathbf{0} & \mathbf{0} & \mathbf{0} & \mathbf{0} & \mathbf{23.47} \end{matrix}\right]$ | --- | --- |
| Piezoelectric coupling Matrix (C/m^2^) | $\left[\begin{matrix} \mathbf{0} & \mathbf{0} & \mathbf{0} & \mathbf{0} & \mathbf{17.03} & \mathbf{0} \\ \mathbf{0} & \mathbf{0} & \mathbf{0} & \mathbf{17.03} & \mathbf{0} & \mathbf{0} \\ \mathbf{-6.62} & \mathbf{-6.62} & \mathbf{23.24} & \mathbf{0} & \mathbf{0} & \mathbf{0} \end{matrix}\right]$ | --- | --- |
